# A cluster randomised controlled trial to assess the effectiveness of a multi-strategy sustainability intervention on teachers’ sustained implementation of classroom physical activity breaks (energisers): study protocol

**DOI:** 10.1186/s12889-023-16810-5

**Published:** 2023-10-07

**Authors:** Nicole Nathan, Alix Hall, Adam Shoesmith, Adrian E. Bauman, Belinda Peden, Bernadette Duggan, Carly Gardner, Cassandra Lane, Christophe Lecathelinais, Christopher Oldmeadow, Craig Duncan, Daniel Groombridge, Edward Riley-Gibson, Emma Pollock, James Boyer, John Wiggers, Karen Gillham, Martina Pattinson, Megan Mattingly, Nicole McCarthy, Patti-Jean Naylor, Penny Reeves, Philippa Budgen, Rachel Sutherland, Rebecca Jackson, Thomas Croft, William Pascoe, Luke Wolfenden

**Affiliations:** 1 Hunter New England Population Health, Hunter New England Area Health Service, Newcastle, NSW Australia; 2 School of Medicine and Public Health, The University of Newcastle, Newcastle, NSW Australia; 3 National Centre of Implementation Science, Hunter New England Area Health Service, Newcastle, NSW Australia; 4 Hunter Medical Research Institute, New Lambton Heights, NSW Australia; 5 Hunter New England Population Health, Hunter New England Local Health District, Locked Bag No. 10, Wallsend, NSW 2287 Australia; 6 Prevention Research Collaboration, Sydney School of Public Health, Sydney, Australia; 7 Charles Perkins Centre (D17), The University of Sydney, Sydney, NSW 2006 Australia; 8 Faculty of Medicine and Health, The University of Sydney, Camperdown, NSW Australia; 9 Catholic Schools Office Diocese of Maitland-Newcastle, Newcastle, NSW Australia; 10 The NSW Department of Education, Sydney, NSW Australia; 11 Health Promotion, Northern NSW Local Health District, Lismore, NSW Australia; 12 Health Promotion, Murrumbidgee Local Health District, Suite 1B/620 Macauley Street, Albury, NSW 2640 Australia; 13 School of Exercise Science, Physical and Health Education, University of Victoria, Victoria, BC Canada; 14 Health Promotion Service, Illawarra Shoalhaven Local Health District, NSW Health, Warrawong, NSW Australia; 15 Aboriginal Health Unit, Hunter New England Local Health District, Wallsend, NSW Australia

**Keywords:** Sustainability, Sustainment, Implementation science, School policy, Physical activity, Energiser, Schools

## Abstract

**Background:**

Governments internationally have invested hugely in the implementation and scale-up of school-based physical activity interventions, but have little evidence of how to best sustain these interventions once active implementation support ceases. This study will assess the effectiveness of a multi-strategy sustainability intervention on classroom teachers’ sustainment of energisers (short 3–5 min physical activity breaks during class-time) scheduled across the school day from baseline to 12 and 24-month follow-up.

**Methods:**

A cluster randomised controlled trial will be conducted in 50 primary schools within the Hunter New England, Illawarra Shoalhaven, Murrumbidgee and Northern New South Wales (NSW) Local Health Districts of NSW Australia. Schools will be randomly allocated to receive either usual support or the multi-strategy sustainability intervention that includes: centralised technical assistance from a trained project officer; formal commitment and mandated change obtained from school principals; training in-school champions; reminders for teachers; educational materials provided to teachers; capturing and sharing local knowledge; and engagement of parents, carers and the wider school community. The primary trial outcome will be measured via a teacher logbook to determine the between-group difference in the change in mean minutes of energisers scheduled across the school day at 12 and 24-month follow-up compared to baseline. Analyses will be performed using an intention to treat framework. Linear mixed models will be used to assess intervention effects on the primary outcome at both follow-up periods.

**Discussion:**

This study will be one of the first randomised controlled trials to examine the impact of a multi-strategy sustainability intervention to support schools’ sustainment of a physical activity intervention. The proposed research will generate new evidence needed for the partnering organisations to protect their considerable investments to date in physical activity promotion in this setting and will provide seminal evidence for the field globally.

**Trial registration:**

ACTRN12620000372987 version 1 registered 17^th^ March 2020. Version 3 (current version) updated 4^th^ August 2023.

**Supplementary Information:**

The online version contains supplementary material available at 10.1186/s12889-023-16810-5.

## Introduction

To support children to meet daily physical activity guidelines the World Health Organization (WHO) has recommended schools implement physical activity policies that promote and enable children’s regular physical activity throughout the day [1, 2]. Accordingly, many countries have developed policies or guidelines mandating the minimum amount of physical activity schools are to provide students each week. For example, the United Kingdom [3] and parts of Canada [4] and the United States [5] require schools to schedule between 120–150 min per week weekly physical activity. In New South Wales (NSW), Australia, the Department of Education (DoE) requires schools to schedule at least 150 min of planned moderate, with some vigorous, physical activity across the school week for students in Kindergarten to Grade 10 [6]. This can include time scheduled for physical education (PE), sport or other structured activities including integrated lessons and “energisers” (i.e., short 3–5 min physical activity breaks during class-time) [6]. Despite the existence of these policies, international research suggests that only 30% of schools routinely implement them [7–13].

The application of implementation science methods has led to significant improvements in schools’ implementation of physical activity interventions [14]. For example, a 2022 Cochrane review of six randomised controlled trials (RCTs) aimed at assessing the effectiveness of strategies to enhance the implementation of school physical activity policies and practices found significant improvements in intervention schools relative to control (standardised mean difference 1.53, 95% CI: 0.78 to 2.28, I^2^ = 85.6%) [14]. Among these, a model of implementation support, developed by our research team, has been found to be effective at increasing schools’ implementation of the NSW school physical activity policy [6]. The multi-strategy implementation intervention (Physically Active Children in Education (PACE)) has demonstrated, across a series of RCTs, to increase teachers’ scheduling of physical activity by 36–44 min per week [13, 15, 16]. Across all studies, teacher’ scheduling of energisers was consistently found to be driving the intervention effect, with increases of approximately 23 min of energisers scheduled each week and contributing to 52–70% of the increase in overall weekly physical activity scheduled [13, 15, 16].

Maximising the benefits and health impact of these school physical activity interventions requires their sustained implementation. Sustainability is defined as: *“after a defined period of time, a program, clinical intervention, and/or implementation strategies continue to be delivered and/or individual behaviour change (i.e., clinician, patient) is maintained; the program and individual behaviour change may evolve or adapt while continuing to produce benefits for individuals/systems”* [17]. Achieving sustainability is however a considerable challenge. A comprehensive review of 125 empirical studies of public health interventions reported less than 23% of programs were sustained at least two years following initial implementation [18]. Specifically within schools, a recent review of the sustainability of health behaviour interventions found that of the 18 included school-based interventions, none were sustained in their entirety following withdrawal of initial implementation support [19]. Of concern, reviews suggest that when programs are not sustained, prevalence typically reverts to pre-intervention levels (or below) [20] and can adversely impact stakeholder trust and willingness to engage in future initiatives [18, 21].

Schools face a number of barriers to sustaining health promoting interventions [19, 22, 23]. For example, a recent review of the determinants of schools’ sustainment of chronic disease prevention interventions found that the most frequent barriers include: the availability of funding, equipment, resources and facilities, continued executive or leadership support, staff turnover and workforce shortages, competing priorities, perceived program effectiveness or benefit and adaptability of the intervention [23]. There is however, little evidence of the most effective strategies to support schools to overcome these barriers and sustain their implementation of health promoting interventions [24]. Systematic reviews in school and childcare settings conducted by the research team have failed to identify any interventions to achieve sustained program implementation of interventions to promote physical activity or other health behaviours [25]. Furthermore, whilst a number of theoretical frameworks for sustainability exist [26, 27], their capacity to effectively support sustained program implementation remains largely untested [21]. As a result, policy makers and practitioners responsible for the promotion of physical activity in schools lack evidence to support sustainment of evidence-based interventions (EBI). Therefore, the primary aim of this trial is to assess the effectiveness of a multi-strategy sustainability intervention on classroom teacher sustainment of minutes of energisers scheduled across the school day from baseline to 12- and 24-month follow-up.

## Methods

### Trial registration and ethical approval

This study will be employed using a National Health and Medical Research Council (NHMRC) Partnership grant and coordinated by Hunter New England (HNE) Population Health staff and University of Newcastle researchers. It was prospectively registered with the Australian New Zealand Clinical Trials Registry (ACTRN12620000372987 – registered 17th March 2020) and has Human Research Ethics approval from HNE Human Research Ethics Committee (no. 2019/ETH12353), the University of Newcastle Human Research Ethics Committee (no. H-2008–0343), NSW Department of Education (no. 2017184) and the relevant Catholic Schools Offices. The study methods will be reported in accordance with the Consolidated Standards of Reporting Trials (CONSORT) statement for cluster RCTs [28], and the Standards for Reporting Implementation Studies (StaRI) statement [29]. See Supplementary file 1 for the completed Standard Protocol Items: Recommendations for Intervention Trials (SPIRIT) checklist.

### Design and setting

This study will employ a parallel group cluster RCT design. Classroom teachers from primary schools within the HNE, Illawarra Shoalhaven, Murrumbidgee and Northern NSW Local Health Districts (LHDs), of NSW Australia that have recently (within the last 12 months) received the PACE implementation strategy will be invited to participate. Consenting schools will be randomised to receive a multi-strategy sustainability intervention to support the continued implementation of energisers across the school day; or usual care. The trial will assess between-group differences in the average change in mean daily minutes of energisers implemented, with data collected at baseline (November 2022 to October 2023), 12 months post-baseline immediately following the delivery of the sustainability strategy (November 2023 to October 2024) and 24-months post-baseline (November 2024 to October 2025) (see Table 1 for a detailed trial timeline).

**Table 1:**
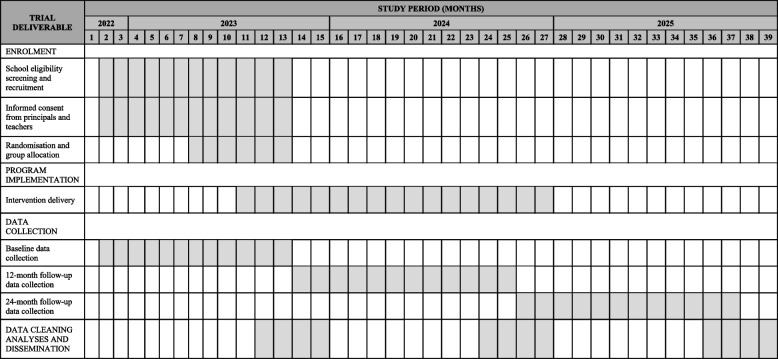
Timeline of school enrolment, data collection and intervention delivery

### Participants and recruitment

*Schools:* Eligible schools will include all government, Catholic and independent (private) primary schools that have received *Physically Active Children in Education (PACE*) training to support their implementation of weekly physical activity, including energisers. Schools will be excluded if they are participating in another physical activity intervention or cater exclusively for children with special needs. Principals from eligible schools will be provided with a study information package and asked to provide online written informed consent.

*Teachers:* All classroom teachers from eligible and consenting schools will be randomised to receive the sustainability strategy and an invitation to participate in data collection. Strategies will be employed to ensure high recruitment rates are achieved, including delivering a series of two follow-up emails and a phone call reminder to schools [30]. To maximise principal and teacher survey participation rates, evidence-based strategies will be delivered, including sending two email reminders to teachers (one week apart) and one follow-up phone call reminder to In-school Champions (ISCs) to complete the survey, as well as distribution of a AUD$30 online gift card from a national grocery store for each principal and teacher who completes a survey in gratitude for their time. To maximise the retention of schools over the study period, strategies will be enacted including the delivery of non-program resources (including a pen, post-it notepad, and stickers for each class), to each teacher during data collection, as well as a letter of appreciation from the project team outlining the broad positive impact of their participation [31–33]. Previous studies conducted by the research team utilising such strategies have yielded school and or teacher participation rates of > 80% and attrition of < 20% [13, 34, 35].

### Randomisation and blinding

An independent statistician will use a computerised random number function to randomise schools in a 1:1 ratio to either an intervention or control group. Randomisation will occur following consent and baseline data collection to reduce the risk of selection bias. Block randomisation will ensure group allocation is approximately equal. Allocation will not be stratified by any school-level factor given a lack of clear prognostic factors for the sustainability of physical activity implemented in schools [36, 37]. However, schools will be stratified by time (school phase) and by LHD (jurisdiction) to ensure the allocation across each LHD is approximately equal. Due to the nature and delivery of the intervention, school staff and program delivery staff will become aware of school group allocation. Data analysts will be blinded.

### Intervention group: Multi-strategy sustainability intervention

The 12-month multi-strategy sustainability intervention was co-developed with a trial Advisory Group consisting of health and education policy makers, health promotion practitioners, teachers, physical activity experts, implementation and behavioural scientists, education and public health practitioners. The development process was guided by formative evaluation undertaken by the research team to identify determinants to sustaining school based physical activity interventions generally and energisers specifically. Specifically this involved: i) systematic review evidence of sustainability determinants within the school setting [23]; ii) quantitative surveys with 240 classroom teachers assessing factors associated with local sustainability of weekly physical activity implementation using the adapted Program Sustainability Assessment Tool [37, 38]; and iii) qualitative research from our previous implementation trials (school-based observations by program delivery personnel, and interviews, and surveys of school staff [13, 39]. Utilising this evidence, identified barriers were mapped to the Integrated Sustainability Framework [21]. This empirically informed sustainability-determinants framework was developed for application in the field of public health to identify and synthesise multi-level factors previously found to influence the sustainability of EBIs across a range of community settings, including schools [21]. Barriers were also mapped to the Behaviour Change Wheel (BCW) [40] and Theoretical Domains Framework (TDF) [41] to ensure: i) consideration of a comprehensive assessment of factors (i.e., capabilities, opportunities and motivation) impacting on an individual’s behaviour; and ii) identification of modifiable factors and potential behaviour change techniques that may be utilised to influence or enact the desired behaviour of an individual to sustain practice change. Potential sustainability strategies and behaviour change techniques were then identified using the recommended process described by Michie et al. [40] and, in consultation with members of the Advisory Group, assessed against the APEASE criteria [42] for their Affordability, Practicality, Effectiveness and cost effectiveness, Acceptability, Side-effects/safety and Equity. Finally, strategies were aligned to the sustainment-explicit Expert Recommendations for Implementing Change (ERIC) glossary [43], to ensure consistency in nomenclature, definitions and use of strategies. Table 2 includes a detailed description of each of the sustainability strategies using the sustainment-explicit ERIC Glossary [43] and how the delivery of each strategy will be operationalised according to the Action, Actor, Context, Target, Time AACTT) framework [44]; and shows how strategies were mapped against the Integrated Sustainability Framework, BCW and TDF to address barriers and behaviours to sustaining teacher’s daily scheduling of energisers.

**Table 2:** Description of sustainability strategies mapped to the relevant theories and taxonomies

**Sustainability strategy according to Nathan et al. **[43]	**Proposed mechanism of action**				**Intervention content**	
	**Barriers addressed**	**Integrated Sustainability Framework domain**	**COM-B and (TDF)**	**Intervention functions**	**BCT Behaviours**	**Sustainability strategy detailed explanation (according to the AACTT) framework **[44]
**Centralize technical assistance and provide ongoing consultation**	• Teachers knowledge, ability or competence • Lack of time • Perceived priority of the program in the schools	• Inner contextual factors • Characteristics of the interventionist and population	• Psychological capability (beliefs about capabilities; knowledge) • Social opportunity (environmental context and resources) • Reflective motivation (goals)	• Enablement • Persuasion	• Review behaviour goal(s) • Review outcome goal(s)	Project officers (a PE teacher and health promotion practitioner) will provide technical assistance to schools throughout the study period to support the sustained scheduling of daily energisers. They will work directly with schools and ISC to overcome barriers and provide expertise support and resources Project officers will provide ongoing consultation to ISCs via telephone or email to support the delivery of sustainability strategies
**Obtain formal commitments**	• Lack of executive endorsement and leadership • Changing priorities • Lack of support from executive and other staff • Limited school culture/identity	• Characteristics of the interventionist and population • Inner contextual factors	• Reflective motivation (beliefs about capabilities) • Physical opportunity (environmental context and resources) • Social opportunity (social influences) • Reinforcement	• Enablement • Environmental restructuring	• Social support • Reframing • Professional / social role and identity	Signed executive commitment will be obtained by project officers during an in-person meeting with the principal at the start of the study period, pledging their support to staff in the scheduling of daily energisers. Principals will commit to support their staff to continue to scheduling energisers by: • Encouraging teachers to schedule daily energisers by forwarding an email of support to all staff • Sharing evidence that energisers are an easy and effective way to help meet the NSW 150-min physical activity policy • Allocating a regular short agenda item in staff meetings to share ideas and discuss the delivery of energisers • Displaying their school’s ‘Energiser School’ fence sign, framed certificate and newsletter snippets • Completing a whole school physical activity timetable that indicates > 80% of classes schedule energisers 5 days a week
**Mandate change**	• Limited belief in their ability to schedule in a crowded curriculum (when competing priorities arise) • Limited dedicated time to and within schedule • Changing priorities; external and school • Lack of executive endorsement and leadership	• Characteristics of the interventionist and population • Inner contextual factors • Characteristics of the intervention	• Reflective motivation (beliefs about capabilities) • Physical opportunity (environmental context and resources) • Social opportunity (social influences)	• Enablement • Environmental restructuring	• Social support • Reframing • Prompts and cues	Following obtaining signed formal commitment, schools will receive a physical ‘Energiser School’ certificate and a fence sign to display reflecting to staff and the school community the school’s commitment to scheduling daily energisers. Principals will also receive a modifiable email template from project officers that can be personalised and circulated to their staff further highlighting their support to schedule daily energisers by: • Scheduling energisers every day • Contacting their ISC or project officer for support • Sharing their ideas at staff meetings • Accessing the PACE online portal for resources and ideas
**Identify and prepare champions**	• Lack of time in the curriculum • Teachers knowledge, ability or competence	• Inner contextual factors • Characteristics of the interventionist and population	• Social opportunity (environmental context and resources) • Psychological and physical capability (beliefs about capabilities)	• Modelling • Education • Training	• Identification of self as role model • Social support (unspecified) • Problem solving • Instruction on how to perform a behaviour • Demonstration of the behaviour	Each school will nominate up to three ISCs at the start of the study period (existing teachers at the school who were previously identified and supported the initial implementation of PACE) to facilitate delivery of the sustainability strategies within each school. ISCs have previously undergone training to prepare them for their role (i.e., 1-day [5-h] face-to-face workshop run by project officers) and received a program delivery manual outlining their role, how they can support their staff, and where to seek program support Project officers will provide support to ISCs to overcome resistance that the program may provoke in the school, or navigate any barriers faced to sustaining the daily scheduling of energisers
**Remind teachers**	• Forgetting to schedule each day • Lack of variety/adaptability of energisers • Limited access to energiser resources	• Characteristics of the interventionist and population	• Psychological capability (memory, attention and decision processes)	• Enablement	• Prompts and cues • Social support • Credible source • Instruction on how to perform the behaviour	Project officers will send targeted email prompts to ISCs quarterly, at the beginning of each school term. Each email will: • Prompt ISCs to remind classroom teachers to schedule daily energisers • Include a link for teachers to access the existing online resource repository • Act as a mechanism to identify turnover in ISCs, by asking them to confirm that they are still available and have capacity to act in this role • Include a link to video testimonials or information snippets to include in their school newsletter, with the purpose of endorsing the scheduling of daily energisers, highlighting benefits of energisers and how to navigate any emerging barriers or priorities faced
**Distribute educational materials**	• Remembering to schedule each term • Knowing what are energisers and which ones to do • Belief in their ability to schedule in a crowded curriculum (when competing priorities arise) • School culture/identify as an energiser school • Boredom with resources and scheduling • Limited adaptability and variety of resources • Limited accessible resources • Feeling overwhelmed • Staff turnover • Lack of support from executive • Changing priorities	• Inner contextual factors • Processes • Characteristics of the interventionist and population • Characteristics of the intervention	• Psychological capability (memory, attention and decision processes) • Reflective motivation (beliefs about capabilities) • Reinforcement • Emotion • Physical opportunity (environmental context and resources) • Social opportunity (social influences)	• Enablement • Environmental restructuring • Modelling • Persuasion • Professional / social role and identity • Incentivisation • Training	• Adding objects to the environment • Social support • Prompts/cues • Reframing • Demonstration of the behaviour • Information about social and environmental consequences • Information about health consequences • Social comparison • Self-monitoring of behaviour	Project officers will develop training and educational materials to disseminate to classroom teachers in the form of an energiser resource pack following receipt of formal commitment obtained from the school principal. The resources will be designed to remind teachers to schedule energisers each day and will include: • A template classroom timetable highlighting where teachers could potentially schedule energisers throughout the school day • An erasable A3 whiteboard sign where teachers can record their daily energisers for the class to see • An infographic with a QR code to the online portal of resources • An infographic linking common barriers to sustaining the delivery of energisers with possible solutions • A lanyard displaying a range of energiser options including the names and ‘how to’ deliver each to their class New ISCs will also be sent a number of resources that will help upskill them and become familiar with the program, their role and where to seek program support including: (i) a copy of the ISC manual (electronic and hard copy); and (ii) online access to the online portal training modules for ISCs. This will be sent whenever a new ISC is identified within the school
**Involve parents consumers and family members**	• Limited belief in benefits – for teachers and students • Lack of leadership support and endorsement • Lack of parental support	• Characteristics of the interventionist and population • Characteristics of the intervention	• Reflective motivation (beliefs about consequences) • Social opportunity (social influences)	• Persuasion • Environmental restructuring	• Information about health consequences • Credible source • Information about others approval • Restructuring the social environment	A range of documents will be provided by project officers and used to communicate to parents and the wider school community the school’s dedication to scheduling daily energisers, and to illustrate to parents and carers the health, social, and learning benefits of energisers. These documents will include: • Information snippets embedded in the existing email prompts sent each school term that schools can include in their newsletter, online webpage or communications channel; and • An information leaflet for inclusion in the kindergarten orientation booklet during Term 3 of the school year
**Capture and share local knowledge**	• Lack of belief in their ability to schedule in a crowded curriculum (when competing priorities arise) • Limited belief in benefits – for teachers and students	• Characteristics of the interventionist and population • Characteristics of the intervention	• Reflective motivation (beliefs about capabilities) • Beliefs about consequences	• Enablement • Modelling • Persuasion	• Reframing • Demonstration of the behaviour • Information about social and environmental consequences • Information about health consequences	Video testimonials of a classroom teacher and principal/executive who have previously taken part in PACE and continue to schedule energisers in their school will be available on the online portal and embedded in the existing email prompts (that will be sent each school term by project officers to ISCs for distribution to other teachers within their school). These testimonials will highlight the importance of delivering energisers, and showcase the range of benefits, adaptability/flexibility and solutions to overcome barriers faced within the school

### Control group and contamination

The delivery of all intervention components will be under the control of the research team and will not be provided to control group schools during the intervention period. Schools in the control group will receive ‘usual’ (reactive) support which is provided to schools as part of existing service delivery within the respective LHD. This involves the provision of information and resources specific to the PACE program on the existing online portal and includes factsheets, example policies, and templates not related to sustainability. According to evidence and theory [45], such strategies do not address the primary obstacles to sustainability, thus any impact on the primary trial outcome is likely to be minimal. Nonetheless, data regarding schools’ exposure to potential sources of contamination (or co-intervention) will be assessed via items included in teacher and principal follow-up surveys – if evident, potential effects on outcomes will be explored via sensitivity analyses.

### Data collection and management

Principal and teacher data will be obtained through self-report surveys. These will be administered as a pen-and-paper version or using the Hunter New England Population Health (HNEPH) instance of the online data capture tool Research Electronic Data Capture (REDCap) [46, 47], depending on their preference. Management of trial data will be in accordance with a data management protocol, which has been developed and approved by the Advisory Group. Data will be stored securely as per the requirements of the HNE Human Research Ethics Committee and The University of Newcastle Human Research Ethics Committee. Data will only be accessible to primary researchers and statisticians. Confidential participant data will be stored securely and not linked to survey responses.

### Measures

All outcome measures will be collected at baseline, 12- and 24-months post-recruitment.

### Primary trial outcome

#### Sustainment of mean minutes of daily energisers scheduled by classroom teachers over 12- and 24-months

Given the scheduling of energisers consistently possessed the greatest impact on teachers overall scheduling of weekly physical activity in our previous implementation trials [13, 16], and thus increased the likelihood of achieving adherence of the NSW policy [6]; the primary trial outcome will be the between-group difference in the change in mean minutes of energisers scheduled across the school day at 24 month follow-up compared to baseline [18]. Outcome data will also be collected at 12 months in order to describe attenuation patterns. Scheduled daily energisers for each class will be assessed via classroom teacher completion of a daily activity log-book for one full school week (5-days). At the end of each school day during the week of data collection, each teacher will complete a log of the time and occasions they planned physical activity for: energisers, PE, Sport or other structured activities e.g., integrated lessons. The use of teacher logbooks is frequently used in classroom-based obesity prevention interventions, with high response rates (i.e. > 80%) [48, 49] and established reliability [50]. This outcome measure and data collection method has been used in our previous studies assessing teachers’ scheduling of classroom physical activity, with completion rates of ~ 88% [13, 16, 51], which is the premise for use in the current study.

### Secondary trial outcomes

#### Sustainment of mean minutes of weekly physical activity (PE, sport and other structured activities) scheduled by classroom teachers over 12- and 24-months

The difference in the change in mean minutes of overall physical activity and the individual components that make up overall physical activity, including sport and PE, and other planned activities (e.g., active lessons and energisers) implemented by classroom teachers across the school week, assessed via logbooks completed by teachers at 12 and 24-month follow-up compared to baseline.

#### Difference in the change in proportion of school adherence to the 150 min physical activity policy from baseline to 12- and 24-month follow-up

The difference between groups in the change in proportion of schools, from baseline to 12- and 24-month follow-up adhering to the government policy of 150 min of scheduled classroom activity per week.

#### Sustainability determinants to teachers’ scheduling of daily energisers

To assist in understanding the determinants experienced and addressed in this trial we will assess the theoretical factors hypothesised to impact the sustainability of teacher implementation of energisers [21]. Specifically, principals and classroom teachers will complete newly developed measures theoretically informed by the Integrated Sustainability Framework [21] to assess the determinants of sustaining school-based public health interventions at baseline, 12- and 24-months. Each measure was designed to assess the factors perceived by respective stakeholders (executive-level e.g., principal or executive staff member and implementer-level e.g., classroom teacher) as influential to the sustainability of EBIs delivered in the school setting. Using a five-point scale (1 = not at all influential; 2 = slightly influential; 3 = moderately influential; 4 = extremely influential; and 5 = not applicable to me), principals and teachers will be asked to indicate how much the listed factors influence the delivery of daily energisers at their school. The 28-item executive scale to be completed by principals covers four framework domains and focuses on higher organisational and structural-level factors e.g., socio-political context, external funding, external partnerships. Whereas the 42-item implementer scale to be completed by classroom teachers covers all five framework domains and examines factors more relevant to frontline intervention delivery e.g., motivation, capability, training, executive support, and available resources.

### Implementation outcomes

To characterise sustainment, the measures recommended by Proctor et al. [52] of implementation outcomes will also be assessed. This includes;

- *Acceptability –* The perceived acceptability of each sustainability support strategy will be assessed via a paper or online-based survey completed by principals and classroom teachers of intervention schools at 12 and 24-month follow-up using items from the validated Acceptability of Intervention Measure developed by Weiner et al. [53].
- *Adoption –* Based upon a previously developed tool from the research team [13, 54], at 12- and 24-month follow-up all intervention and control principals will be asked to report, via paper or online based survey, their school’s adoption for scheduling energisers each day (i.e., proportion of classes at each school scheduling energisers each day).
- *Appropriateness –* The perceived appropriateness of each sustainability support strategy will be assessed via a paper or online-based survey completed by principals and classroom teachers of intervention schools at 12- and 24-month follow-up using items from the validated Intervention Appropriateness Measure developed by Weiner et al. [53].
- *Feasibility –* The perceived feasibility of each sustainability support strategy will be assessed via a paper or online-based survey completed by principals and classroom teachers of intervention schools at 12- and 24-month follow-up using items from the validated Feasibility of Intervention Measure developed by Weiner et al. [53].
- *Fidelity –* Project records as well as post-intervention questionnaires completed by intervention principals, ISC, and teachers will be used to determine the proportion of schools that received and attended to each of the sustainability strategies.
- *Strategy implementation cost –* Defined as the cost impact of sustainability effort; see economic analysis section below.
- *Penetration –* This will be calculated, using scheduling data from the teacher survey at 12- and 24-month follow-up, as the number of teachers who schedule daily energisers per school, divided by the total number of teachers expected to schedule daily energisers [52].
- *Sustainability –* See primary outcome section above.

### Other measures

#### Economic analysis

A prospective economic analysis measuring the incremental cost and outcomes of the sustainability strategies will be undertaken from adapted societal and health service perspectives. Resource use for the intervention and usual practice will be prospectively identified and measured from project records (staff and consumables). Direct costs associated with the intervention are anticipated to include labour (sustainability support), and program materials. Systems to prospectively log and document costs were developed for our previous trials [13, 14, 39, 51] and will be adapted to the proposed study. Incremental cost will be calculated as the difference between intervention and usual care cost. The primary outcome for the economic analysis will align to the trial outcome, which is the between-group difference in the change in mean minutes of energisers scheduled across the school day at 24 months follow-up compared to baseline. A ‘within trial’ economic analysis will assess program value by comparing incremental costs and benefits at the school level across the study arms. Uncertainty intervals will be calculated for the mean incremental cost result and incremental cost effectiveness ratio using non-parametric bootstrapping. Resource use measurement will occur prospectively and continuously over the duration of the trial.

#### School characteristics and process data

Data regarding the operational characteristics of schools will be collected using a combination of surveys of the school website as well as survey items completed by school principals and classroom teachers that we have used in previous studies [13, 16]. Project officer records and survey items will be used to record delivery of sustainability support strategies, and exposure of individual schools and teachers to such strategies. Data will be collected, stored, and managed on the HNEPH server using REDCap [46, 47].

### Statistical analysis

All analyses will be undertaken under an intention-to-treat framework. Analyses of outcomes at 12-month follow-up will provide evidence of any immediate impact of the intervention. The 24-month follow-up will provide evidence of sustainment of energiser implementation and will represent the primary end-point. Teachers will be the unit of analysis. Between-group differences in the mean change in the primary outcome will be assessed at each time-point using linear mixed models. Models will include fixed effects for treatment group (intervention vs control), time (baseline, 12-month, and 24-month follow-up), and a time-by-treatment group interaction term. A random level intercept for school will be included to account for the clustered design of the study. A random intercept for teacher nested within school will also be included to account for potential repeated measures by teachers. The linear mixed models will use all available data, assuming missing data is at random.

### Sample size

We are aiming for a sample size of 40 schools (20 per arm). Assuming a comparator mean of 7.08 min of energisers scheduled daily, a standard deviation of 4.88, an average of five classes per school and an intraclass correlation coefficient (ICC) of 0.11, based on our previous trial [13], a sample of 40 schools is sufficient to detect (with 80% power, an alpha of 0.05) a mean difference of approximately 2.38 min of daily energisers.

### Research trial governance

This study has employed a research co-production approach in its design [55]. An external multi-disciplinary Advisory Group, consisting of 16 members including education policy makers, health promotion practitioners, Aboriginal Health Officers, as well as researchers with expertise in physical activity, implementation science, behavioural science, education and public health will oversee all aspects of the planning, implementation and evaluation of the project. A project team consisting of research staff and practitioners will conduct the study according to study protocol. The Advisory Group will oversee the project dissemination plan including all publications and reports to stakeholders. Authorship will conform to the International Committee of Medical Journal Editors guidelines.

### Trial discontinuation or modification

Our Advisory Group will convene once a quarter to ensure the study is abiding by the prescribed ethics and timeline. It is not anticipated that any events would occur that warrant discontinuing the trial. However, any unforeseen adverse events will be recorded and assessed by the trial Advisory Group and reported to the HNE Human Research Ethics Committee (primary approval committee), with advice sought regarding required action. The trial registration record will be updated with any protocol modifications, and any deviations from original protocol will be reported in study outcome papers.

## Discussion

Physical inactivity is a leading cause of death and disability in Australia and internationally [56, 57] and is identified as a priority health issue [1, 2]. Improving children’s activity levels is key to reducing the development of both short and long-term health burdens [58, 59]. School-based physical activity policies effectively improve child physical activity levels [50, 60, 61]. However, sustaining their implementation remains a considerable challenge globally [18, 19]. In Australia alone, approaches to sustain the implementation of school-based policies that mandate minimum periods for structured physical activity have the potential to improve the health, well-being and chronic disease risk of two million students [62]. This study is one of the first RCTs to test the effectiveness and efficiency of theoretically and empirically informed strategies to improve the sustainability of an EBI targeting a chronic disease risk factor in schools. The proposed trial will be seminal for the field, translate into fundamental outcomes in the knowledge base of sustainability research, and provide a platform for future research examining the sustainability of effective EBIs in the school setting.

### Supplementary Information

**Additional file 1.** SPIRIT 2013 Checklist: Recommended items to address in a clinical trial protocol and related documents

## Data Availability

All study materials are available from the research team upon request to lead investigators.

## Abbreviations

AACTT: Action, Actor, Context, Target, Time
APEASE: Affordability, Practicality, Effectiveness and cost effectiveness, Acceptability, Side-effects/safety and Equity
BCW: Behaviour Change Wheel
CONSORT: Consolidated Standards of Reporting Trials
DoE: Department of Education
ERIC: Expert Recommendations for Implementing Change
EBI: Evidence-based intervention
HNE: Hunter New England
ICC: Intraclass correlation coefficient
ISC: In-school champion
MVPA: Moderate-to-vigorous physical activity
NHMRC: National Health and Medical Research Council
NSW: New South Wales
PACE: Physically Active Children in Education
PE: Physical Education
RCT: Randomised controlled trial
REDCap: Research Electronic Data Capture
StaRI: Standards for Reporting Implementation Studies
TDF: Theoretical Domains Framework

## References

[1] WHO (2020). Guidelines on physical activity and sedentary behaviour..

[2] Bull FC, Al-Ansari SS, Biddle S, Borodulin K, Buman MP, Cardon G (2020). World Health Organization 2020 guidelines on physical activity and sedentary behaviour. Br J Sports Med online.

[3] UK Chief Medical Officers (2019). UK Chief Medical Officers' Physical Activity Guidelines.

[4] Canadian Society for Exercise Physiology (2019). Canadian 24-Hour Movement Guidelines for Children and Youth: An Integration of Physical Activity, Sedentary Behaviour, and Sleep.

[5] U.S. Department of Health and Human Services. Physical Activity Guidelines for Americans. 2nd ed. Washington, DC: Department of Health and Human Services; 2018.

[6] NSW Government. Rationale for change; sport and physical activity policy- revised 2015. In: NSW Department of Education and Communities, ed. Sydney: School Sport Unit; 2015.

[7] Carlson JA, Sallis JF, Chriqui JF, Schneider L, McDermid LC, Agron P (2013). State policies about physical activity minutes in physical education or during school. J Sch Health.

[8] Thompson HR, Linchey J, Madsen KA (2013). Peer Reviewed: Are Physical Education Policies Working? A Snapshot from San Francisco, 2011. Prev Chronic Dis.

[9] Harrington DM, Belton S, Coppinger T, Cullen M, Donnelly A, Dowd K (2014). Results from Ireland’s 2014 Report Card on Physical Activity in Children and Youth. J Phys Act Health.

[10] Olstad adoption, diffusion, implementation and impact of provincial daily physical activity policies in Canadian schools. BMC Public Health. 2015; 15:385. 10.1186/s12889-015-1669-6. 10.1186/s12889-015-1669-6 PMC4436021 25885026

[11] Johansen DLN, Christensen BFN, Fester M (2018). Results from Denmark’s 2018 Report Card on Physical Activity for Children and Youth. J Phys Act Health.

[12] Mâsse LC, Naiman D, Naylor PJ (2013). From policy to practice: implementation of physical activity and food policies in schools. Int J Behav Nutr Phys Act.

[13] Nathan N, Hall A, McCarthy N, Sutherland R, Wiggers J, Bauman AE, et al. Multi-strategy intervention increases school implementation and maintenance of a mandatory physical activity policy: outcomes of a cluster randomised controlled trial. Br J Sports Med. 2022;56:385–93. 10.1136/bjsports-2020-103764 PMC8938653 34039583

[14] Wolfenden L, McCrabb S, Barnes C, O'Brien KM, Ng KW, Nathan NK, et al. Strategies for enhancing the implementation of school‐based policies or practices targeting diet, physical activity, obesity, tobacco or alcohol use. Cochrane Database of Systematic Reviews. 2022, Issue 8. Art. No.: CD011677. 10.1002/14651858. 10.1002/14651858.CD011677.pub3 PMC9422950 36036664

[15] Nathan N, Wiggers J, Bauman AE, Rissel C, Searles A, Reeves P (2019). A cluster randomised controlled trial of an intervention to increase the implementation of school physical activity policies and guidelines: study protocol for the physically active children in education (PACE) study. BMC Public Health.

[16] Nathan NK, Sutherland RL, Hope K, McCarthy NJ, Pettett M, Elton B (2020). Implementation of a School Physical Activity Policy Improves Student Physical Activity Levels: Outcomes of a Cluster-Randomized Controlled Trial. J Phys Act Health.

[17] Moore JE, Mascarenhas A, Bain J, Straus SE (2017). Developing a comprehensive definition of sustainability. Implement Sci.

[18] Wiltsey Stirman S, Kimberly J, Cook N, Calloway A, Castro F, Charns M (2012). The sustainability of new programs and innovations: a review of the empirical literature and recommendations for future research. Implement Sci.

[19] Herlitz L, MacIntyre H, Osborn T, Bonell C (2020). The sustainability of public health interventions in schools: a systematic review. Implement Sci.

[20] Shediac-Rizkallah MC, Bone LR (1998). Planning for the sustainability of community-based health programs: conceptual frameworks and future directions for research, practice and policy. Health Educ Res.

[21] Shelton RC, Cooper BR, Stirman SW (2018). The Sustainability of Evidence-Based Interventions and Practices in Public Health and Health Care. Annu Rev Public Health.

[22] Cassar S, Salmon J, Timperio A, Naylor PJ, van Nassau F, Contardo Ayala AM (2019). Adoption, implementation and sustainability of school-based physical activity and sedentary behaviour interventions in real-world settings: a systematic review. Int J Behav Nutr Phys Act.

[23] Shoesmith A, Hall A, Wolfenden L, Shelton RC, Powell BJ, Brown H (2021). Barriers and facilitators influencing the sustainment of health behaviour interventions in schools and childcare services: a systematic review. Implement Sci.

[24] Hailemariam M, Bustos T, Montgomery B, Barajas R, Evans LB, Drahota A (2019). Evidence-based intervention sustainability strategies: a systematic review. Implement Sci.

[25] Wolfenden L (2019). What happens once a program has been implemented? A call for research investigating strategies to enhance public health program sustainability. Aust N Z J Public Health..

[26] Hodge LM, Turner KMT (2016). Sustained implementation of evidence-based programs in disadvantaged communities: a conceptual framework of supporting factors. Am J Community Psychol.

[27] Luke DA, Calhoun A, Robichaux CB, Elliott MB, Moreland-Russell S (2014). The program sustainability assessment tool: a new instrument for public health programs. Prev Chronic Dis.

[28] Campbell MK, Piaggio G, Elbourne DR, Altman DG, Group ftC (2012). Consort 2010 statement: extension to cluster randomised trials. BMJ..

[29] Pinnock H, Barwick M, Carpenter CR (2017). Standards for Reporting Implementation Studies (StaRI) Statement. BMJ (Clinical research ed).

[30] Wolfenden L (2012). Acceptability of proactive telephone recruitment to a telephone support service to encourage healthy eating, physical activity and weight loss. Aust N Z J Public Health..

[31] Brueton V, Stenning SP, Stevenson F, Tierney J, Rait G (2017). Best practice guidance for the use of strategies to improve retention in randomized trials developed from two consensus workshops. J Clin Epidemiol.

[32] Drews, K., Harrell, J., Thompson, D. et al. Recruitment and retention strategies and methods in the HEALTHY study. Int J Obes. 2009;33(4):S21–S28 (2009). 10.1038/ijo.2009.113 10.1038/ijo.2009.113 PMC2758033 19623184

[33] Teague S, Youssef GJ, Macdonald JA, Sciberras E, Shatte A, Fuller-Tyszkiewicz M (2018). Retention strategies in longitudinal cohort studies: a systematic review and meta-analysis. BMC Med Res Methodol.

[34] Sutherland R (2017). A trial of an intervention to facilitate the implementation of school-based practices known to increase students’ MVPA. Am J Prev Med..

[35] Nathan N (2016). Effectiveness of a multicomponent intervention to enhance implementation of a healthy canteen policy in Australian primary schools: A RCT. Int J Behav Nutr Phys Act..

[36] McIntosh K, Mercer SH, Nese RNT (2018). Factors predicting sustained implementation of a universal behavior support framework. Educ Res.

[37] Shoesmith A, Hall A, Wolfenden L. et al. School-level factors associated with the sustainment of weekly physical activity scheduled in Australian elementary schools: an observational study. BMC Public Health. 2022; 22, 1408. 10.1186/s12889-022-13732-6 10.1186/s12889-022-13732-6 PMC9308175 35870895

[38] Hall A, Shoesmith A, Shelton RC (2021). Adaptation and validation of the program sustainability assessment tool (PSAT) for use in the elementary school setting. Int J Environ Res Public Health.

[39] Lane C, Wolfenden L, Hall A, Sutherland R, Naylor PJ, Oldmeadow C (2022). Optimising a multi-strategy implementation intervention to improve the delivery of a school physical activity policy at scale: findings from a randomised noninferiority trial. Int J Behav Nutr Phys Act.

[40] Michie S, van Stralen MM, West R (2011). The behaviour change wheel: a new method for characterising and designing behaviour change interventions. Implement Sci.

[41] Atkins L, Francis J, Islam R, O'Connor D, Patey A, Ivers N (2017). A guide to using the Theoretical Domains Framework of behaviour change to investigate implementation problems. Implement Sci.

[42] Michie S, Atkins LRW (2014). The Behaviour Change Wheel: A Guide To Designing.

[43] Nathan N, Powell BJ, Shelton RC, Laur CV, Wolfenden L, Hailemariam M (2022). Do the Expert Recommendations for Implementing Change (ERIC) strategies adequately address sustainment?. Front Health Serv.

[44] Presseau J, McCleary N, Lorencatto F, Patey AM, Grimshaw JM, Francis JJ. Action, actor, context, target, time (AACTT): a framework for specifying behaviour. Implement Sci. 2019;14(102). 10.1186/s13012-019-0951-x 10.1186/s13012-019-0951-x PMC6896730 31806037

[45] Birken SA, Haines ER, Hwang S, Chambers DA, Bunger AC, Nilsen P. Advancing understanding and identifying strategies for sustaining evidence-based practices: a review of reviews. Implement Sci. 2020;15(88). 10.1186/s13012-020-01040-9. 10.1186/s13012-020-01040-9 PMC7545853 33036653

[46] Harris PA, Taylor R, Thielke R, Payne J, Gonzalez N, Conde JG (2009). Research electronic data capture (REDCap)–a metadata-driven methodology and workflow process for providing translational research informatics support. J Biomed Inform.

[47] Harris PA, Taylor R, Minor BL, Elliott V, Fernandez M, O'Neal L, et al; REDCap Consortium. The REDCap consortium: Building an international community of software platform partners. J Biomed Inform. 2019;95:103208. 10.1016/j.jbi.2019.103208. 10.1016/j.jbi.2019.103208 PMC7254481 31078660

[48] Bessems KM, Van Assema P, Martens MK, Paulussen TG, Raaijmakers LG, De Vries NK (2011). Appreciation and implementation of the Krachtvoer healthy diet promotion programme for 12- to 14- year-old students of prevocational schools. BMC Public Health.

[49] van Nassau F, Singh AS, van Mechelen W, Paulussen TG, Brug J, Chinapaw MJ (2013). Exploring facilitating factors and barriers to the nationwide dissemination of a Dutch school-based obesity prevention program "DOiT": a study protocol. BMC Public Health.

[50] Cradock AL (2014). Impact of the Boston Active School Day Policy to Promote Physical Activity among Children. Am J Health Promot..

[51] Hall A, Wolfenden L, Shoesmith A, McCarthy N, Wiggers J, Bauman AE (2022). The impact of an implementation intervention that increased school's delivery of a mandatory physical activity policy on student outcomes: A cluster-randomised controlled trial. J Sci Med Sport.

[52] Proctor E, Silmere H, Raghavan R (2011). Outcomes for implementation research: conceptual distinctions, measurement challenges, and research agenda. Admin Pol Ment Health.

[53] Weiner BJ, Lewis CC, Stanick C (2017). Psychometric assessment of three newly developed implementation outcome measures. Implement Sci.

[54] Reilly KL, Nathan N, Wiggers J, Yoong SL, Wolfenden L (2018). Scale up of a multi-strategic intervention to increase implementation of a school healthy canteen policy: findings of an intervention trial. BMC Public Health.

[55] Wolfenden L, Yoong SL, Williams CM (2017). Embedding researchers in health service organizations improves research translation and health service performance: the Australian Hunter New England Population Health example. J Clin Epidemiol..

[56] Kohl HW (2012). The pandemic of physical inactivity: global action for public health. Lancet..

[57] Katzmarzyk PT, Friedenreich C, Shiroma EJ, Lee IM (2022). Physical inactivity and non-communicable disease burden in low-income, middle-income and high-income countries. Br J Sports Med.

[58] Craigie AM, Lake AA, Kelly SA, Adamson AJ, Mathers JC (2011). Tracking of obesity-related behaviours from childhood to adulthood: A systematic review. Maturitas.

[59] Poitras VJ, Gray CE, Borghese MM, Carson V, Chaput JP, Janssen I (2016). Systematic review of the relationships between objectively measured physical activity and health indicators in school-aged children and youth. Appl Physiol Nutr Metab.

[60] Pate RR, Trilk JL, Byun W, Wang J (2011). Policies to increase physical activity in children and youth. J Exerc Sci Fit.

[61] Brennan LK, Brownson RC, Orleans CT (2014). Childhood obesity policy research and practice: evidence for policy and environmental strategies. Am J Prev Med.

[62] Robertson-Wilson JE (2012). Physical activity policies and legislation in schools: a systematic review. Am J Prev Med.

